# Ultrasensitive Vibrational Imaging of Retinoids by Visible Preresonance Stimulated Raman Scattering Microscopy

**DOI:** 10.1002/advs.202003136

**Published:** 2021-02-08

**Authors:** Minghua Zhuge, Kai‐Chih Huang, Hyeon Jeong Lee, Ying Jiang, Yuying Tan, Haonan Lin, Pu‐Ting Dong, Guangyuan Zhao, Daniela Matei, Qing Yang, Ji‐Xin Cheng

**Affiliations:** ^1^ State Key Laboratory of Modern Optical Instrumentation College of Optical Science and Engineering Zhejiang University Hangzhou 310027 China; ^2^ Department of Biomedical Engineering Boston University Boston MA 02215 USA; ^3^ College of Biomedical Engineering and Instrument Sciences Zhejiang University Hangzhou 310027 China; ^4^ Department of Electrical and Computer Engineering Boston University Boston MA 02215 USA; ^5^ Department of Chemistry Boston University Boston MA 02215 USA; ^6^ Department of Obstetrics and Gynecology Northwestern University Feinberg School of Medicine Chicago IL 60611 USA; ^7^ Robert H. Lurie Comprehensive Cancer Center Chicago IL 60611 USA; ^8^ Collaborative Innovation Center of Extreme Optics Shanxi University Taiyuan 030006 China; ^9^ Photonics Center Boston University Boston MA 02215 USA

**Keywords:** fingerprint, retinoids, stimulated Raman scattering, ultrasensitive, visible preresonance

## Abstract

High‐sensitivity chemical imaging offers a window to decipher the molecular orchestra inside a living system. Based on vibrational fingerprint signatures, coherent Raman scattering microscopy provides a label‐free approach to map biomolecules and drug molecules inside a cell. Yet, by near‐infrared (NIR) pulse excitation, the sensitivity is limited to millimolar concentration for endogenous biomolecules. Here, the imaging sensitivity of stimulated Raman scattering (SRS) is significantly boosted for retinoid molecules to 34 micromolar via electronic preresonance in the visible wavelength regime. Retinoids play critical roles in development, immunity, stem cell differentiation, and lipid metabolism. By visible preresonance SRS (VP‐SRS) imaging, retinoid distribution in single embryonic neurons and mouse brain tissues is mapped, retinoid storage in chemoresistant pancreatic and ovarian cancers is revealed, and retinoids stored in protein network and lipid droplets of *Caenorahbditis elegans* are identified. These results demonstrate VP‐SRS microscopy as an ultrasensitive label‐free chemical imaging tool and collectively open new opportunities of understanding the function of retinoids in biological systems.

## Graphical abstract

By shifting the excitation laser wavelength to approach the absorption of retinol, visible preresonance stimulated Raman scattering (VP‐SRS) microscope achieves an unprecedented 34 µm detection sensitivity. VP‐SRS microscope reveals retinoid distribution in embryonic neurons and mouse brain tissues, discovers retinoid storage in chemoresistant cancers, and identifies retinoids stored inside *Caenorahbditis elegans*.

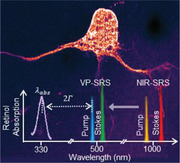

Retinoids, or vitamin A derivatives, are one of the essential nutrients for animals. Mammals are not able to synthesize retinoids de novo, and therefore must uptake from dietary. Retinoids play crucial roles in embryonic development,^[^ ^1^ ^]^ immunity,^[^ ^2^ ^]^ stem cell differentiation^[^ ^3^ ^]^ and lipid metabolism,^[^ ^4^ ^]^ and their deficiency leads to a variety of human disorders.^[^ ^5^ ^]^ Aldehyde dehydrogenase 1 (ALDH1), as a detoxifying enzyme functioning in converting retinol into retinoic acid,^[^ ^6^ ^]^ is overexpressed in several drug‐resistant cancer cells.^[^ ^7^ ^]^

A variety of tools have been used to probe retinoids. With high specificity and sensitivity, high‐performance liquid chromatography and mass spectroscopy are widely used to measure retinoids extracted from a bulk sample.^[^ ^8^ ^]^ However, the ensemble measurement without spatial‐temporal information renders difficulties to map retinoid distribution within a cell and understand the corresponding functions. Spontaneous Raman spectromicroscopy directly probes biomolecules in a label‐free manner. However, the small Raman scattering cross‐section limits the pixel dwell time at second‐scale in order to accumulate enough photons.^[^ ^9^ ^]^ Coherent Raman scattering (CRS) microscopy, based on coherent anti‐Stokes Raman scattering or stimulated Raman scattering (SRS), significantly boosts the signal level and thus allows for in situ imaging of metabolites in living cells.^[^ ^10^, ^11^ ^]^ CRS microscopy has been used to map glucose metabolism,^[^ ^12^, ^13^, ^14^ ^]^ cholesterol storage,^[^ ^15^, ^16^, ^17^ ^]^ fatty acid metabolism,^[^ ^18^, ^19^, ^20^, ^21^ ^]^ triglyceride,^[^ ^22^ ^]^ nucleic acid,^[^ ^23^, ^24^ ^]^ protein aggregates,^[^ ^25^ ^]^ and delivered small molecules.^[^ ^26^, ^27^, ^28^ ^]^ Thus far, with imaging sensitivity at millimolar level for endogenous biomolecules, CRS microscopy is only applied to retinoid‐rich droplets in liver tissue^[^ ^29^ ^]^ and *Caenorahbditis elegans*.^[^ ^30^ ^]^ In order to resolve retinoid metabolic heterogeneity at subcellular level, ultrasensitive in situ imaging of retinoids is desired.

Electronic preresonance SRS significantly enhances the sensitivity by applying pump wavelength close to the electronic absorption wavelength with detuning of *ω*_abs_ − *ω*_pump_ ≈ 2Γ (where Γ is the homogeneous linewidth). Several efforts have been made to enable preresonance SRS imaging based on Raman tag synthesis and protein engineering to shift the absorption band toward near‐infrared (NIR) window where the laser pulses reside (Figure S1, Supporting Information). Using this strategy, Wei et al. increased the detection sensitivity of Raman tags by conjugating vibrational probes with NIR dyes for preresonance SRS enhancement.^[^ ^31^ ^]^ Lee et al. demonstrated membrane voltage imaging via NIR preresonance SRS probing of red‐shifted microbial rhodopsins expressed in *Escherichia coli*.^[^ ^32^ ^]^

Here, we demonstrate a different strategy of preresonance SRS imaging by shifting the excitation laser wavelength to approach the absorption of intrinsic chromophores (**Figure** **1**A). Specifically, we convert the pump and Stokes wavelengths to visible region through frequency doubling. In an earlier study, Wang and co‐workers have shown visible‐wavelength SRS imaging^[^ ^33^ ^]^ at a spatial resolution of 130 nm and a spectral resolution of 26 cm^−1^. However, its potential for high‐sensitivity preresonance imaging is yet to be explored. In this work, we report a visible preresonance SRS (VP‐SRS) microscope for imaging of retinoids with an unprecedented 34 × 10^−6^ m detection sensitivity at 10 µs pixel dwell time, which is shorter by 10^5^ times than the time needed for NIR‐SRS detection of 50 × 10^−6^ m retinoids.^[^ ^34^ ^]^ By spectral focusing, we achieved 10 cm^−1^ spectral resolution, which allows hyperspectral imaging of multiple biomolecules in fingerprint region. With such capacity, we explored chemically selective mapping of retinoids in embryonic neurons, whole mouse brain, chemoresistant cancer cells, and protein network in *C. elegans*, which is beyond the reach by the widely used NIR‐CRS microscope.^[^ ^29^, ^30^ ^]^

**Figure 1:**
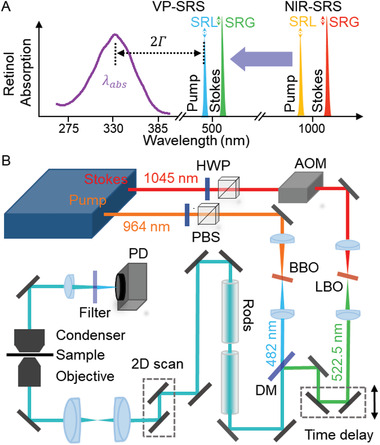
VP‐SRS microscope. A) Diagram of ultrasensitive detection of retinol by electronic preresonance SRS at visible wavelengths. B) Schematic of the VP‐SRS microscope. HWP: half wave plate. PBS: polarizing beamsplitter. AOM: acousto‐optical modulator. BBO: beta‐barium borate. LBO: lithium borate. DM: dichroic mirror. PD: photodiode.

The schematic of our VP‐SRS microscope is shown in Figure 1B. In brief, two NIR laser lines at wavelength of 964 and 1045 nm are frequency‐doubled by a barium borate (BBO) and a lithium triborate (LBO) crystal, respectively, to generate visible pump and Stokes beams at 482 and 522.5 nm. The Stokes beam is modulated by an acousto‐optic modulator (AOM) before the LBO crystal. Instead of an optical fiber,^[^ ^33^ ^]^ we deploy a pair of rods to chirp both beams. The combined beams are sent into a lab‐built microscope. An oil immersion objective with 1.49 numerical aperture (N.A.) is used to focus the laser and an oil condenser to collect the signal photons. The pump beam passes through a filter and is detected by a photodiode with a resonant circuit at 2.2 MHz. As the pump wavelength 482 nm approaches the tail of retinoid absorption profile with ≈325 nm peak wavelength and ≈3500 cm^−1^ line width (Figure S2, Supporting Information), a preresonance regime (*ω*_abs_ − *ω*_pump_  ≈  2Γ) is reached.

We have characterized the performance of our system using well‐defined specimens. First, to evaluate the sensitivity enhancement by preresonance and laser frequency doubling, we compared the signal to noise ratio (SNR) of the same retinol solution imaged by NIR‐SRS and VP‐SRS microscopes. In **Figure** **2**A, the SNR of retinol solution diluted with dimethyl sulfoxide (DMSO) is ≈15 under NIR‐SRS with the 10 mW pump power and 22.5 mW Stokes power, whereas the SNR of the same sample is ≈380 under VP‐SRS with the 2.25 mW pump power and 6.25 mW Stokes power. After correcting the power differences, the effective SNR improvement is 190 times by shifting the laser excitation from NIR to visible wavelength region. This number shows a good agreement with the theoretical calculation discussed further in detail. Second, to determine the ultimate detection sensitivity for retinoids, we imaged retinol solutions diluted with DMSO (Figure 2B). The lowest detectable concentration is 34 × 10^−6^ m under the imaging condition of 10 µs pixel dwell time with 2.89 mW pump power and 15 mW Stokes power at the sample (Figure S3, Supporting Information). The detection sensitivity is at least two orders of magnitude higher compared with previously published record of 50 × 10^−6^ m retinol under condition of 1 s pixel dwell time.^[^ ^34^ ^]^ Third, to evaluate the spatial resolution of the VP‐SRS microscope, we imaged 75 nm polystyrene beads at ≈3050 cm^−1^ peak (Figure 2C). The cross‐section profile of one selected bead is indicated in Figure 2D. By fitting with Gaussian function, the measured full width at half maximum (FWHM) is ≈126 nm. After deconvolution with the actual size of the bead, we determined our measured point spread function to be ≈111 nm, corresponding to a lateral spatial resolution of ≈133 nm defined by Rayleigh criterion. This number is consistent with the theoretical lateral resolution of 130 nm, derived from $\frac{0.61\;\lambda_{\mathrm{em}}}{\sqrt{2}\;N.A.}$, where *λ*_em_ denotes the wavelength of the visible SRS excitation laser as 450 nm; N.A. is 1.49. Fourth, to evaluate the spectral resolution, we acquired a VP‐SRS spectrum targeting the cholesterol peak at 1670 cm^−1^ (sterol C=C bond) as shown in the upper panel of Figure 2E. The FWHM of the 1670 cm^−1^ peak is found to be 9.5 cm^−1^ by Lorentz fitting, which is comparable to the FWHM as 8 cm^−1^ measured by spontaneous Raman spectroscopy, as shown in the lower of Figure 2E. Such high spectral resolution allows us to distinguish chemicals including olive oil, cholesterol, bovine serum albumin (BSA), retinol solution, retinoic acid solution, and retinal solution using their vibrational fingerprints (Figure 2F) within 300 cm^−1^ bandwidth of the VP‐SRS system (Figure S4, Supporting Information). Except retinoid spectra showing transient absorption (TA) background due to the closer absorption to the excitation beam, all VP‐SRS spectra in Figure 2F present good agreements with both the spontaneous Raman and the NIR‐SRS spectra (Figure S5, Supporting Information). The TA‐subtracted SRS signal of retinoids by TA Gaussian fitting is shown in Figure S6 of the Supporting Information.

**Figure 2:**
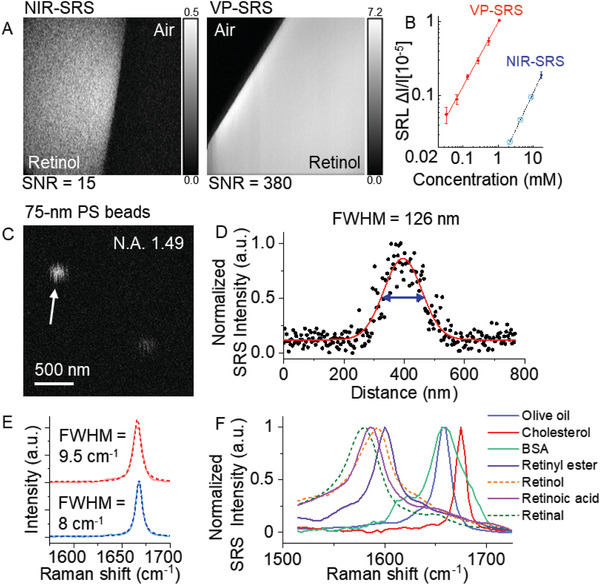
Detection sensitivity, spatial resolution, and spectral resolution of VP‐SRS microscope. A) Imaging 17.45 × 10^−3^ m retinol solution by NIR‐SRS and VP‐SRS for SNR comparison. B) Linear dependence of VP‐SRS signals (red solid dot) on concentrations of retinol diluted in DMSO at 1580 cm^−1^ with 10 µs pixel dwell time. Detection limit of 34 × 10^−6^ m retinol is achieved. One concentration of retinol at 17.45 × 10^−3^ m under NIR‐SRS (blue solid dot) is also depicted, in which SNR is ≈15. Error bars, mean ± s.d. Three lower concentrations are predicted (light blue cross in circle) in the graph. C) VP‐SRS imaging of 75 nm polystyrene beads at ≈3050 cm^−1^ by using an N.A .1.49 oil immersion objective. D) Cross‐section profile of the selected bead indicated by the white arrow in (C) The profile is fitted with a Gaussian function with the fitting curve shown in red. The FWHM of intensity profile is calculated to be 126 nm. E) Upper: VP‐SRS spectrum of cholesterol (red solid line). The Lorentz fitting (red dotted line) gives the bandwidth of the cholesterol peak at 1670 as 9.5 cm^−1^ FWHM. Lower: Spontaneous Raman spectrum of cholesterol (blue solid line). The Lorentz fitting (blue dotted line) gives the bandwidth of the cholesterol peak at 1670 cm^−1^ as 8 cm^−1^ FWHM. F) VP‐SRS spectra of olive oil, cholesterol, BSA, retinyl ester solution, retinol solution, retinoic acid solution, and retinal solution.

Retinoids play an important role in cell differentiation. In particular, retinoic acid, a metabolic product of vitamin A, induces the differentiation of stem cells into a variety of neural cell types by activating the transcription of genes.^[^ ^35^ ^]^ Retinoic acid also induces axon regeneration.^[^ ^36^ ^]^ Conventional way for retinoid detecting is to measure indirectly by targeting the retinoid binding proteins.^[^ ^37^, ^38^ ^]^ Here, we demonstrate in situ retinoid imaging in embryonic neurons.

We first employed the hyperspectral VP‐SRS imaging in a day in vitro (DIV)‐2 embryonic neuron at the fingerprint region from 1500 to 1725 cm^−1^. In **Figure** **3**A, we observed VP‐SRS signal of soma and axon by summing up the VP‐SRS intensity in the fingerprint window. In the VP‐SRS spectrum of neuron, signal at ≈1580 cm^−1^ is assigned to be the signature peak of retinoids (Figure 3B). Signal at 1600–1650 cm^−1^ is assigned to the mixture of C=C bond in retinoids and amide I band in protein, and signal at 1655 cm^−1^ to the mixture of acyl C=C bond in lipids and amide I band in protein. We also compared the retinoid imaging of neurons by VP‐SRS with imaging by NIR‐SRS. In the NIR‐SRS spectrum of neurons, a small peak at ≈1580 cm^−1^ was observed, indicating the presence of retinoids in neurons. For the VP‐SRS, a much significant peak at ≈1580 cm^−1^ was observed, demonstrating the high‐sensitivity retionid imaging by VP‐SRS (Figure S7, Supporting Information).

**Figure 3:**
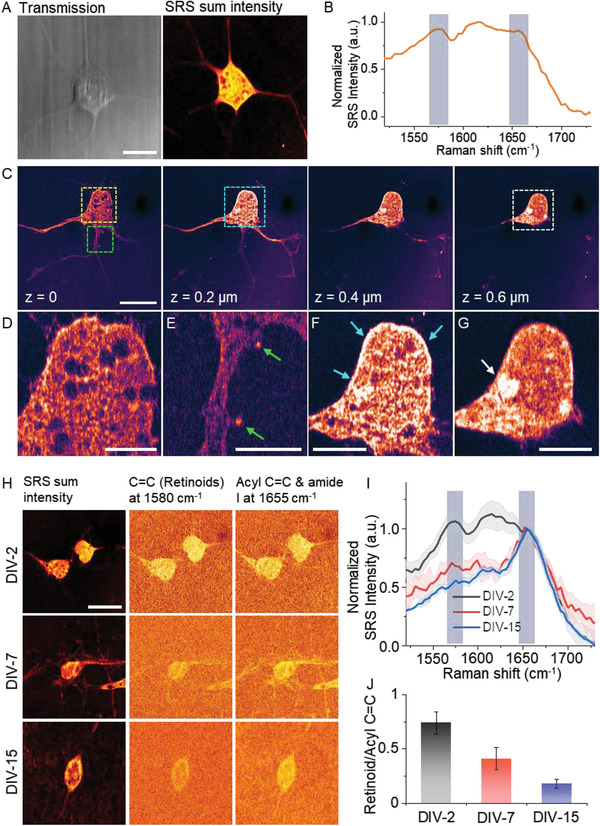
VP‐SRS reveals heterogeneous distribution of retinoids in embryonic neurons at different stages. A) The transmission and SRS sum intensity images of a DIV‐2 embryonic neuron. B) SRS spectrum of neuron in (A). C) 3D SRS imaging of a DIV‐2 embryonic neuron at 1580 cm^−1^. D–G) Zoom‐in images of the area in yellow (D), green (E), blue (F), and white squares (g), marked in (C). H) SRS sum intensity images (left), SRS image of retinoid C=C bond at 1580 cm^−1^ (middle), and SRS image of the mixture of acyl C=C bond and amide I band at 1655 cm^−1^ (right), of the DIV‐2 (upper), DIV‐7 (middle), and DIV‐15 (lower) embryonic neurons. I) SRS spectrum of the DIV‐2, DIV‐7, and DIV‐15 embryonic neurons normalized at 1655 cm^−1^. J) Ratio of TA‐subtracted retinoid signal at 1580 cm^−1^ with to mixed signal from acyl C=C and amide I at 1655 cm^−1^ of the DIV‐2, DIV‐7, and DIV‐15 embryonic neurons. Scale bar: (A,C,H) 10 µm, (D–G) 2.5 µm.

To further validate that the signal at ≈1580 cm^−1^ is from retinoids, several control experiments were performed (Figure S8, Supporting Information). First, by culturing neurons in vitamin A free medium, we found much decreased VP‐SRS signal at ≈1580 cm^−1^ compared with the control. Second, we rescued the vitamin A depletion by supplementing retinol. The signal at ≈1580 cm^−1^ increased after the rescue, compared with the group without retinol supplement. These results collectively prove that VP‐SRS is able to map retinoid molecules in neurons by ≈1580 cm^−1^ peak.

In order to map the retinoid distribution inside a neuron, we performed a 3D VP‐SRS imaging of a DIV‐2 embryonic neuron targeting at the retinoid peak of 1580 cm^−1^ (Figure 3C). When the laser was focused to the portion of neuron close to the cover glass surface (*z* = 0), we observed clear structure of soma, axon, and dendrites. The soma signal mainly comes from the plasma membrane since neuron was flatly attached to the cover glass. We observed heterogeneously distributed retinoid structure which is not reported before. Specifically, retinoids are distributed into a web‐like structure with several retinoid‐lacking regions (Figure 3D). We also observed enrichment of retinoids in the dendritic spines, indicated by the green arrows in Figure 3E. This observation suggests retinoids may play an important role in dendritic growth.^[^ ^36^ ^]^ As we focused on an upper plane of the neuron (*z* = 0.2 µm), we observed that the peripheral region of neuron, indicated by blue arrows, produces stronger retinoid signal (Figure 3F). This result suggests that retinoid is rich in the cell membrane, which is reasonable as retinoids are hydrophobic and lipophilic. As we focused further upward (*z* = 0.6 µm), we observed a ring‐shaped nucleus membrane with high signal level (Figure 3G). Notably, we observed some retinoid signal accumulated inside the nucleus as pointed by the white arrow in Figure 3G.

We next investigated the abundance of retinoids during neuronal development by imaging DIV‐2, DIV‐7, and DIV‐15 embryonic neurons (Figure 3H). By integrating the SRS signal from single neurons at different stages, we observed reduction of retinoids as neurons become mature (Figure 3I). The ratio of TA‐subtracted signal from retinal C=C at 1580 cm^−1^ (Figure S9, Supporting Information) to signal from the mixture of acyl C=C bond and amide I band at 1655 cm^−1^ decreases from ≈1 to 0.5 during the neuronal differentiation (Figure 3J). The observation of decreased retinoid amount as neurons become mature suggests that retinoids are essential molecules to trigger neuronal differentiation.^[^ ^39^ ^]^ Collectively, VP‐SRS imaging at high sensitivity and high spatial resolution reveals a heterogeneous distribution of retinoids in single embryonic neurons, and provides a visual evidence of the important role that retinoids play during cell differentiation.

Retinoids are known to regulate gene expression, maintain neuronal plasticity, and modulate cognitive function in brain.^[^ ^40^ ^]^ Mapping retinoids in a complex brain tissue would provide important information of hierarchical organization of retinoids relating to the brain function. Direct imaging of retinoids in a central nervous system is challenging. Below we show that VP‐SRS microscopy is able to map retinoid distribution in a whole brain tissue prepared from a three‐week old mouse.

VP‐SRS imaging of a whole mouse brain tissue was performed by targeting the retinal C=C bond at 1580 cm^−1^ and the mixture of acyl C=C bond and amide I band at 1655 cm^−1^. Heterogeneous distribution of unsaturated fatty acid and retinoids at different brain regions is found (**Figure** **4**A). In particular, the fimbria (Fi) region, which demonstrates a higher unsaturated fatty acid signal due to abundance of nerve fiber bundles, shows distinctive lipid‐rich signal compared to the adjacent caudate putamen (CPu) region and ventral posterior (VP) nucleus region (Figure 4B). The nerve fiber bundles with similar orientation are presented in VP as indicated by the red arrow in Figure 4D, but not in CPu. Interestingly, there are a large number of retinoid‐rich neurons scattering in CPu, which are not present in VP. Zoom‐in images further reveal the heterogeneity of retinoids at subcellular level. For those retinoid‐rich neurons in CPu, retinoids are distributed in the cytoplasm but not in the nucleus as indicated by the white arrow in Figure 4C, whereas for the neurons in VP, retinoids are absent in both nucleus and cytoplasm, and only found in the membrane as indicated by the black arrow in Figure 4D. As shown in Figure 4E, these two types of neurons are also found in the ventromedial nucleus of the hypothalamus (VMH) region (Figure 4F) and dorsomedial nucleus of hypothalamus (DM) region (Figure 4G). Furthermore, a strong retinoid signal is found in the hippocampus (Figure 4H,I), which suggests that retinoids play important roles in brain plasticity in hippocampus.^[^ ^41^ ^]^ To further confirm that the imaging contrast at 1580 cm^−1^ is based on the Raman signal from retinoid, we performed hyperspectral VP‐SRS imaging of those retinoid‐rich neurons at the ventral lateral (VL) nucleus region (Figure S10A,B, Supporting Information) and hippocampus (Figure S10C,D, Supporting Information). Relative higher Raman peak at 1580 cm^−1^ was observed in those retinoid‐rich neurons compared with the signal from nearby fiber bundles. To summarize, VP‐SRS reveals a heterogeneous distribution of retinoids in a whole mouse brain slice for both tissue and cell levels.

**Figure 4:**
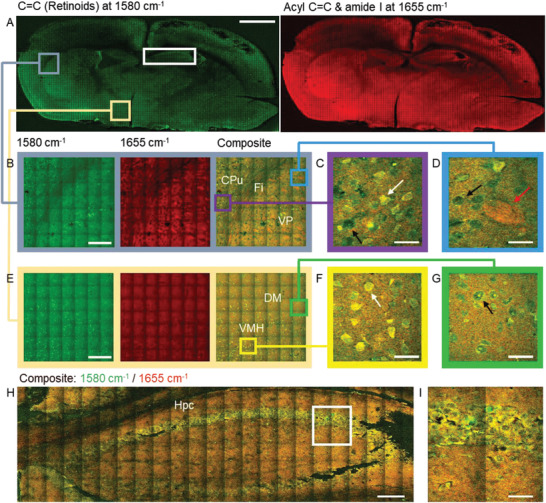
A retinoid map in a whole mouse brain tissue produced by VP‐SRS imaging. A) Large‐area SRS imaging of a whole mouse brain tissue. Left: SRS imaging of C=C bond of retinoids at 1580 cm^−1^; right: SRS imaging of the mixture of acyl C=C bond and amide I band at 1655 cm^−1^. B) Zoom‐in SRS imaging at 1580 cm^−1^ (left), at 1655 cm^−1^ (middle), and composite (right), respectively, of CPu, VP, and Fi marked by the gray square in (A). C) Enlarged view of CPu. D) Enlarged view of VP. E) Zoom‐in SRS imaging at 1580 cm^−1^ (left), at 1655 cm^−1^ (middle), and composite (right), respectively, of VMH and DM marked by the light yellow square in (A). F) Enlarged view of VMH. G) Enlarged view of DM. H) Composite SRS image of hippocampus at 1580 cm^−1^ (retinoids, green) and 1655 cm^−1^ (unsaturated fatty acid, red) marked by the white rectangle in (A). I) Zoom‐in SRS image of the white square in (H). Scale bar: (A) 1 mm, (B,E,H) 100 µm, (C,D,F,G) 15 µm, and (I) 30 µm.

Retinoids are known to play a key role in drug resistance. Overexpression of ALDH1, an enzyme converting retinol into retinoic acid, is observed in multiple chemoresistant cells.^[^ ^7^ ^]^ Nevertheless, intracellular storage and distribution of retinoids in cancer cells is unknown. The unprecedented imaging sensitivity and spatial resolution of VP‐SRS opens the opportunity of mapping and tracing retinoids at subcellular level.

We first performed hyperspectral VP‐SRS imaging of human pancreatic cancer cells, MIA PaCa‐2, at fingerprint region (**Figure** **5**A). We discovered two types of lipid‐rich droplets in MIA PaCa‐2 cells. VP‐SRS imaging of the acyl C=C bond and amide I band at 1655 cm^−1^ clearly reveals the distribution of lipid droplets (LDs) because their composition is primarily triglyceride (Figure 5A, right). As targeting the retinoid peak at 1580 cm^−1^, we observed the retinoid signal is colocalized with a portion of LDs (Figure 5A, middle). Thus, we defined type I LD as the LDs mainly composed of triglyceride, whereas type II LD as the droplets containing triglyceride and retinoids,^[^ ^42^ ^]^ likely in the form of retinyl ester for storage. The representative VP‐SRS spectra of type I and type II LDs indicated by arrows in Figure 5A are shown in Figure 5B. Next, we performed VP‐SRS imaging of two chemoresistant models of pancreatic cancer and ovarian cancer, and quantitatively analyzed the storage of retinoids in chemoresistant versus chemosensitive cancer cells. In the pancreatic cancer model, we used phasor analysis (Figure S11, Supporting Information) to generate type I and II LD mapping based on the signatures of VP‐SRS spectra from both gemcitabine‐sensitive MIA PaCa‐2 cells and gemcitabine‐resistant G3K cells (Figure 5C). The percentage of retinoid‐abundant type II LDs was found to be 98.7% for G3K cells, which was 36.2% higher than that for MIA PaCa‐2 cells (Figure 5D). In the ovarian cancer cell model, we performed VP‐SRS imaging and phasor analysis (Figure S12, Supporting Information) to generate type I and type II LD mapping of cisplatin‐sensitive OVCAR cancer cells and cisplatin‐resistant OVCAR‐cisR cancer cells (Figure 5E). In OVCAR cells, we merely observed type I LDs, whereas in OVCAR‐cisR cells, type I and II LDs were detected to be 72.4% and 27.6% in percentage, respectively (Figure 5F). To validate that the VP‐SRS signal from retinoids, we cultured G3K and MIA PaCa‐2 cells in a medium supplemented with vitamin A. Retinoid signal around 1600 cm^−1^ increased after the vitamin A supplement compared with the control (Figures S13 and S14, Supporting Information). We noted that Raman peak of retinol has been assigned to 1605 cm^−1^.^[^ ^42^ ^]^ The broad distribution of retinoid signal of SRS spectrum from LDs may due to the combination of multiple retinoid formats and the transient absorption signal from retinoids (Figure 2F). The increased percentage of type II LDs both in chemoresistant pancreatic cancer cells and ovarian cancer cells implies a potential important role of retinoid metabolism in drug resistant cancer cells. Our results suggest that VP‐SRS imaging of retinoid‐rich LDs can be used as a label‐free platform to differentiate chemoresistant from chemosensitive cancer cells. Besides, the finding of retinoid storage opens a new opportunity to treat drug‐resistant cancers by targeting the retinoid metabolic pathway.

**Figure 5:**
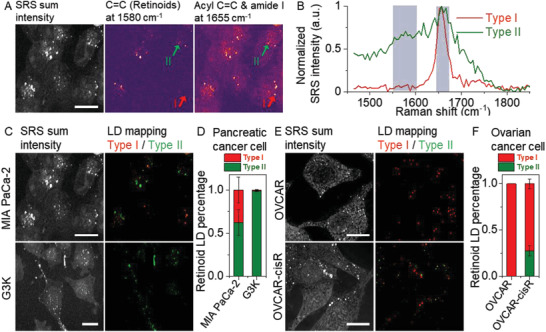
VP‐SRS imaging reveals retinoid storage inside chemoresistant cancer cells. A) SRS imaging of MIA PaCa‐2 cells, (left) by summing up the SRS signal in the fingerprint region, (middle) at 1580 cm^−1^ corresponding to C=C bond of retinoids, (right) at 1655 cm^−1^ corresponding to acyl C=C bond and amide I band. B) SRS spectra of type I and type II LDs indicated by the red and green arrows respectively in (A) normalized at 1655 cm^−1^. C) SRS sum intensity image (left) and LD mapping (right) of MIA PaCa‐2 cells (upper), and G3K cells (lower). D) Statistical analysis of the percentage of type II LDs to all LDs within single cells for MIA PaCa‐2 cells (*N* = 7), and G3K cells (*N* = 7). E) SRS sum intensity image (left) and LD mapping (right) of OVCAR cells (upper), and OVCAR‐cisR cells (lower). F) Statistical analysis of the percentage of type II LDs to all LDs within single cells for OVCAR cells (*N* = 9), and OVCAR‐cisR cells (*N* = 10). *N* is the cell number. Scale bar: 15 µm.

Retinoid homeostasis is essential for gene regulation and survival in living organism, such as *C. elegans*.^[^ ^43^ ^]^ Chen et al., studied retinoid coupling with lipid metabolism in *C. elegans* by applying hyperspectral NIR‐SRS microscopy.^[^ ^30^ ^]^ Here, with much enhanced sensitivity, hyperspectral VP‐SRS microscopy allowed us to probe low‐concentration retinoids in living *C. elegans*. Representative SRS imaging at 1580 cm^−1^ maps the heterogeneous distribution of retionids at C=C bond (**Figure** **6**A); SRS imaging at 1655 cm^−1^ maps the distribution of a mixture of fatty acid and protein at acyl C=C bond and amide I band, respectively (Figure 6B). By plotting SRS spectra from zoom‐in regions (Figure 6C,D), we observed that the blue squared region is rich in retinoids, indicated by the signature peak of retionids at 1580 cm^−1^, along with protein, indicated by relative broader peak at 1655 cm^−1^ (Figure 6E); whereas nearby green squared region obtains fatty acid indicated by a narrower peak at 1655 cm^−1^ (Figure 6E). Besides, the mixed peak, signaling from retinoids and protein at C=C bond and amide I band, respectively, emerges at ≈1650 cm^−1^. The retinoid‐rich region, distributed in the worm body and distinct from lysosome‐related organelles or lipid‐rich regions, is not reported in the previous NIR‐SRS imaging work.^[^ ^30^ ^]^ In order to map retinoid distribution in *C. elegans*, we performed two‐color, 3D VP‐SRS imaging targeting at 1580 and 1655 cm^−1^ (Figure 6F). Droplets composed with different ratio of fatty acid and retinoids were observed. Specifically, droplets rich in retinoids and fatty acid are indicated by yellow and white arrows, respectively. Collectively, VP‐SRS imaging reveals a broad retinoid distribution in the *C. elegan* body as well as its storage in the form of droplets.

**Figure 6:**
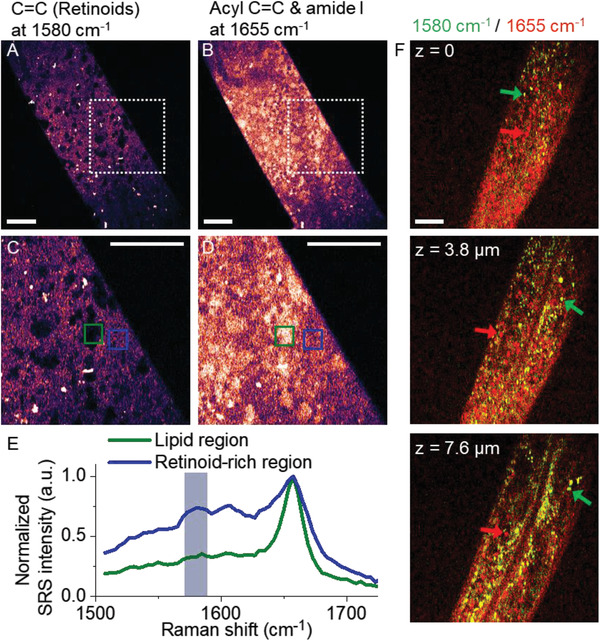
VP‐SRS reveals heterogeneous distribution of retinoids in *C. elegans*. A,B) VP‐SRS images of a living *C. elegan* (A) at 1580 cm^−1^ corresponding to C=C bond of retinoid, and (B) at 1655 cm^−1^ corresponding to a mixture of acyl C=C bond and amide I band. C,D) Zoom‐in VP‐SRS images (C) at 1580 cm^−1^, and (D) at 1655 cm^−1^ of the white dashed square in (A), and (B), respectively. E) SRS spectra of the retinoid‐rich region indicated by the blue square, and LD‐like region from green square in (C), and (D), respectively. The spectra are normalized at 1655 cm^−1^. F) Two‐color, 3D SRS imaging of a *C. elegan* for z‐axis position at 0 (upper), at 3.8 µm (middle), and at 7.6 µm (lower). SRS imaging at 1580 and 1655 cm^−1^ is labeled as green and red, respectively. Scale bar: 10 µm.

Detection sensitivity has been a bottleneck for broad applications of Raman‐based chemical imaging. In 1970s, resonance Raman has been demonstrated as an effective way of boosting the sensitivity.^[^ ^44^ ^]^ Recently, NIR preresonance SRS imaging has been harnessed by a few groups using Raman probes absorption in the NIR window.^[^ ^31^, ^32^ ^]^ In this work, we present a new strategy by shifting the laser wavelength to approach the absorption of endogenous molecules. For retinol, we have reached 34 × 10^−6^ m detection sensitivity at 10 µs pixel dwell time. Using NIR‐SRS, 1 s pixel dwell time was needed to reach 50 × 10^−6^ m detection sensitivity.^[^ ^34^ ^]^

In our method, the signal enhancement arises from both frequency doubling and electronic preresonance. To differentiate the two contributions, we calculated the theoretical preresonance SRS enhancement factor by shifting excitation wavelengths from NIR to visible regime. The stimulated Raman loss can be expressed as Δ*I*_P_∝  − *N*  ×  *σ*_Raman_  ×  *I*_P_  ×  *I*_S_, where Δ*I*_P_ denotes the relative loss in the pump laser, *N* is the number of vibrational bonds in the imaging volume, *σ*_Raman_ is the Raman scattering cross‐section, and *I*_p_, *I*_S_ are the laser intensities of the pump and Stokes beams. *σ*_Raman_ can be further described as

(1) $$\sigma_{\mathrm{Raman}}=K\times \omega_{\mathrm{pump}}\times \omega_{\mathrm{Stokes}}^{3}\times {\left[\frac{\omega_{\mathrm{pump}}^{2}+\;\omega_{e}^{2}}{{\left(\omega_{e}^{2}-\omega_{\mathrm{pump}}^{2}\right)}^{2}}\right]}^{2}$$

where *ω*_pump_ and *ω*_Stokes_ are the frequencies of pump and Stokes beams, respectively; *ω*_e_ is the frequency for electronic transition, and *K* is a constant related to the target biomolecules.^[^ ^31^ ^]^ We noted the enhancement factor of *σ*_Raman_ can be separated as two contributions in the formula: the shorter wavelength effect, and the preresonance effect as pump excitation closer to the absorption. For the case of probing retinoids, *ω*_e_ corresponds to 922 THz (absorption at 325 nm; Figure S2, Supporting Information), *ω*_pump_ corresponds to 622 THz (482 nm), *ω*_Stokes_ corresponds to 574 THz (522.5 nm), targeting vibrational mode corresponds to 1594 cm^−1^. In this electronic preresonant case of probing retinoids, *σ*_Raman_ is calculated to have 158 times increase from shifting NIR‐SRS to visible wavelength SRS. Meanwhile, *I*_P_ can increase by 3.46 times, and *I*_S_ can increase by 4.0 times because of the smaller focal spot. The number of vibrational bonds N decreases by ≈6.44 times due to reduced focal volume. Therefore, the overall signal of VP‐SRS will be increased by 158 × 3.46 × 4 ÷ 6.44 = 339 times compared to NIR‐SRS. For the noise, shot noise is the dominant part of SRS imaging, which is proportional to the square root of photon quantity. Thus, the factor of the sensitivity enhancement should be 462 (Part S2, Supporting Information). Our experiment measurement (VP‐SRS imaging performance) shows that 190 times have been realized which already makes a big difference in retinoid detecting in situ. SHG process made the beam especially the pump beam much narrower, which could be the key barrier.

By shifting the excitation from NIR to visible wavelength, photodamage due to higher photon energy is an issue.^[^ ^33^ ^]^ To minimize photodamage in VP‐SRS, a low laser power and a pixel dwell time as short as 1 µs were used for bioimaging. Video‐rate scanning by the combination of a resonant mirror with a Galvo mirror could further mitigate the heat accumulation in a pixel. This strategy provides a higher photodamage threshold, suggesting the detection sensitivity could be further improved with increased laser power.

We note that by operating SRS excitation at visible wavelength, TA signal or photothermal signal from other molecules inside biosystems is observed. To improve the selectivity, we scanned 30 frames before SRS imaging acquisition to bleach those non‐SRS signals and avoid the interference with our measurement (Figure S15, Supporting Information). The bleached molecules could be cytochrome c with Soret band absorption around 430 nm and Q‐band absorption around 520 nm, which is close to VP‐SRS excitation. Even though the remaining signal is stable after scanning for 30 frames, photodamage to light sensitive molecules should be considered for the sample damage. On the other hand, not all TA or photothermal signal is bleached. Endogenous autofluorescent molecules, i.e., NADH and FAD, may contribute to the non‐SRS background. NADH, reported to be enriched in the LDs,^[^ ^45^ ^]^ may contribute to the broad TA or photothermal background in the VP‐SRS spectrum of LDs in cancer cells. Therefore, we also perform the background fitting in the raw spectra of type II LD both in MIA PaCa‐2 (Figure S16, Supporting Information), OVCAR‐cisR (Figure S17, Supporting Information) cells to extract the retinoid SRS signal.

We also note that by comparing VP‐SRS spectra with spontaneous Raman or NIR‐SRS spectra of retinoid standard samples (Figure 2F; Figure S5, Supporting Information), TA background of retinoids is observed since laser excitation is close to the absorption band of retinoids. To improve the signal‐to‐background ratio, we employed a high Stokes/low pump power to mitigate the TA background while maintaining the SRS signal level.^[^ ^46^ ^]^

By shifting NIR excitation to visible wavelength, the penetration depth is limited by scattering. Therefore, VP‐SRS system is suitable for imaging transparent samples, serving a good candidate for single‐cell analysis with high resolution. For imaging scattered tissue, it is suggested to incorporate optical clearing with VP‐SRS to extend the penetration depth.

In summary, we have shown ultrasensitive mapping of retinoids in single embryonic neurons, a brain slice, cancer cells, and *C. elegans*, which is beyond the reach by NIR CRS.^[^ ^29^, ^30^ ^]^ Moreover, our method is not limited to retinoids. Many drug molecules, including anticancer drugs and antibiotics, have electronic absorption near 400 nm. We expect that VP‐SRS will be a powerful platform to selectively map such drug molecules in cells and tissues at unprecedented detection sensitivity and spatial resolution.

## Experimental Section

##### Visible Preresonance SRS Microscope

A femtosecond (fs) solid‐state laser provided two synchronized outputs as 80 MHz pulsed laser trains (InSight X3, Spectra‐Physics). The 1045 nm output with 4.3 W power and ≈220 fs pulse width served as the Stokes beam. The other output with 2.6 W average power and ≈120 fs pulse at 962 nm was wavelength‐tunable and served as the pump beam. An AOM was used to modulate the Stokes beam at 2.2 MHz with efficiency of ≈50%. The frequency of the Stokes beam was doubled from 1045 to 522.5 nm through focusing at an LBO crystal. The doubling efficiency was ≈10% with 90 mW output power. The pump beam was tuned to 964 nm and focused to a BBO crystal to attain a frequency‐doubled beam of 482 nm. The doubling efficiency was around 10% with 250 mW output power. The pump and Stokes beam were combined with a dichroic mirror (T505lpxr‐UF1, Chroma). The collimated light was guided to a lab‐built laser scanning microscope. A high‐N.A. (N.A. = 1.49) oil objective (UAPON 100XOTIRF; Olympus)) was used to focus the light on the sample. After collection by an oil condenser, the pump beam was filtered by a bandpass filter (470 nm/40 nm) to block the Stokes beam and detected by a photodiode. The modulated Raman signal was amplified by a lab‐built resonant amplifier circuit and extracted by a digital lock‐in amplifier (HF2LI, Zurich Instrument).

##### Sample Preparation

Samples such as olive oil, retinol solution, and *C. elegans* were sandwiched between a glass coverslip (upper) and a slide glass (bottom) by doubled‐sided tape. Samples like polystyrene beads, cells, and tissues were prepared as follows: 1) Beads or cells stuck to a glass coverslip which was then flipped over on a slide glass; 2) Nail polish was used to seal the glass coverslip around its slides. Between the gap of the two glasses, there contained some liquids.

##### Cell Lines and Chemicals

Human pancreatic cancer cell line MIA PaCa‐2 was obtained from the American Type Culture Collection. MIA PaCa‐2 cells were grown in RPMI‐1640, 10% fetal bovine serum (FBS), and 1% penicillin/streptomycin (P/S). Gemcitabine‐resistant G3K cell line was generated from parental MIA PaCa‐2 cells by culture with 1.0 × 10^−6^ m gemcitabine.^[^ ^47^ ^]^ Human ovarian cancer cell line OVCAR5, provided by Dr. Marcus Peter (Northwestern University, Chicago, IL), was mycoplasma negative and authenticated by Short Tandem Repeat analysis. OVCAR5 cells were grown in RPMI‐1640, 10% FBS, 2 × 10^−3^ m l‐glutamine and 1% P/S. Cisplatin‐resistant OVCAR5‐cisR cell line was generated from parental OVCAR5 cells by exposure to three cycles of cisplatin at IC50, followed by cell recovery. The IC50 to cisplatin was 1.3 × 10^−3^ m, 6.4 × 10^−3^ m for the parental and resistant cells, respectively. For maintenance, all cells were cultured at 37 °C in a humidified incubator with 5% CO_2_ supply. Chemicals including avasimibe, gemcitabine used in vitro and in vivo studies were purchased from Selleckchem.com, cisplatin was from Sigma, and FBS was from Thermo Fisher Scientific.

##### Primary Cortical Neurons

Primary cortical neuron cultures were derived from Sprague‐Dawley rats. Briefly, cortices were dissected out from embryonic day 18 (E18) rats of either sex and then digested with papain (0.5 mg mL^−1^ in Earle's balanced salt solution) (Thermo Fisher Scientific) and plated on poly‐d‐lysine coated coverslips. For primary neuron cultures, cells were first plated in Dulbecco's modified Eagle medium (Thermo Fisher Scientific) containing 10% fetal bovine serum (Thermo Fisher Scientific) and 1% GlutaMAXtm (Thermo Fisher Scientific), which was then replaced 24 h later by a feeding medium (Neurobasal medium supplemented with 2% B‐27 (Thermo Fisher Scientific) and 1% GlutaMAXtm (Thermo Fisher Scientific). Thereafter, the medium was replaced every 3 to 4 days until use. Embryonic neurons at different development stages are prepared by fixing after culture for 2 days, 7 days, and 15 days.

##### Brain Tissue Preparation

The mouse brain slice was prepared from a mouse (Jackson Lab) at age 21 days. PBS was used for perfusion, after which formalin was perfused to fix the brain tissue. Then the brain tissue was frozen sectioned at 100 µm thickness.

##### *C. Elegans* Culturing and Handling

*C. elegans* (daf‐2(e1370)) were obtained from the Caenorhabditis Genetics Center. *C. elegans* husbandry and handling were proceeded according to the protocol.^[^ ^48^ ^]^ *C. elegans* were maintained on Nematode Growth Media plates seeded with OP50 *E. coli* and cultured in 20 °C incubator (Tritech research DT2‐ MP‐47L). For VP‐SRS imaging, *C. elegans* were anesthetized with 1% sodium azide, then transformed to a slide glass and sealed by covering a glass coverslip.

##### Statistical Analysis

Results were represented as means +/± SD or as specified.

## Conflict of Interest

The authors declare no conflict of interest.

## Author Contributions

M.Z. and K.‐C.H. designed the overall research with the guide of J.‐X.C. M.Z. and K.C.H designed and built up the VP‐SRS microscope. M.Z. performed the characterizations of the VP‐SRS microscope and directed all the experiments of SRS imaging. H.J.L. designed the experiments on neurons and prepared the whole mouse brain slice. Y.J. cultured the embryonic neurons. Y.T. cultured the pancreatic and ovarian cancer cells. H.L. supported the algorithm of hyperspectral analysis. P.‐T.D. cultured *C. elegans* and prepared the samples. G.Z. and D.M. helped the preparation of some biological samples. Q.Y. provided suggestions on preparation of polystyrene bead samples. M.Z., K.C.H., and J.‐X.C. wrote the manuscript. All authors read and edited the manuscript.

## Supporting information

Supporting Information
