# Supplementary material for: Ultrasensitive Vibrational Imaging of Retinoids by Visible Preresonance Stimulated Raman Scattering Microscopy
Source: Adv Sci (Weinh). 2021 Feb 8;8(9):2003136. doi: 10.1002/advs.202003136 (PMC8097318; doi:10.1002/advs.202003136)
Supplement: Supplementary file 1 — Supporting Information [file ADVS-8-2003136-s001.pdf]

© 2021 Wiley-VCH GmbH

ADVANCED SCIENCE
Open Access

Supporting Information

for *Adv. Sci.,* DOI: 10.1002/advs.202003136

Ultra-sensitive vibrational imaging of retinoids by visible pre-resonance stimulated Raman scattering microscopy

*Minghua Zhuge, Kai-Chih Huang, Hyeon Jeong Lee, Ying Jiang, Yuying Tan, Haonan Lin, Pu-Ting Dong, Guangyuan Zhao, Daniela Matei, Qing Yang, and Ji-Xin Cheng**

Supporting Information

Ultra-sensitive vibrational imaging of retinoids by visible pre-resonance stimulated Raman scattering microscopy

Minghua Zhuge[#], Kai-Chih Huang[#], Hyeon Jeong Lee, Ying Jiang, Yuying Tan, Haonan Lin, Pu-Ting Dong, Guangyuan Zhao, Daniela Matei, Qing Yang, Ji-Xin Cheng[*]

Part 1: Supplementary Figure

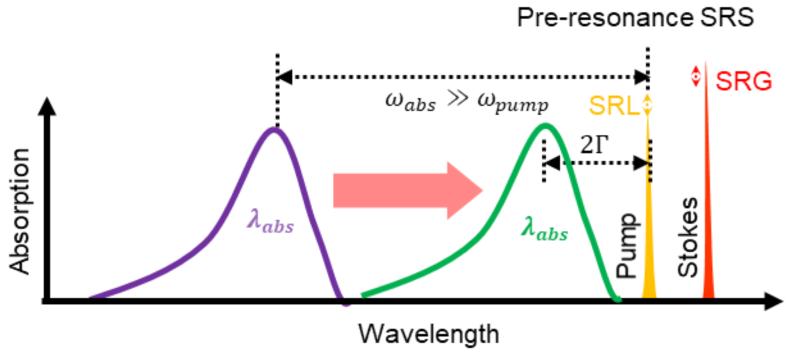

Figure S1. Pre-resonance SRS by probe engineering. Here, the strategy to increase the SRS detection sensitivity is by probe engineering to shift the molecular absorption to approach to the pump wavelength.

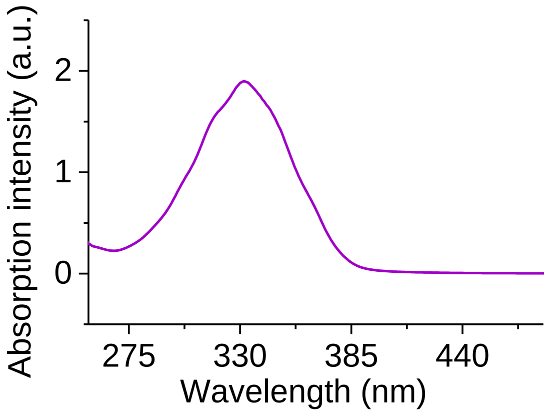

Figure S2. Absorption spectrum of retinol measured in DMSO.

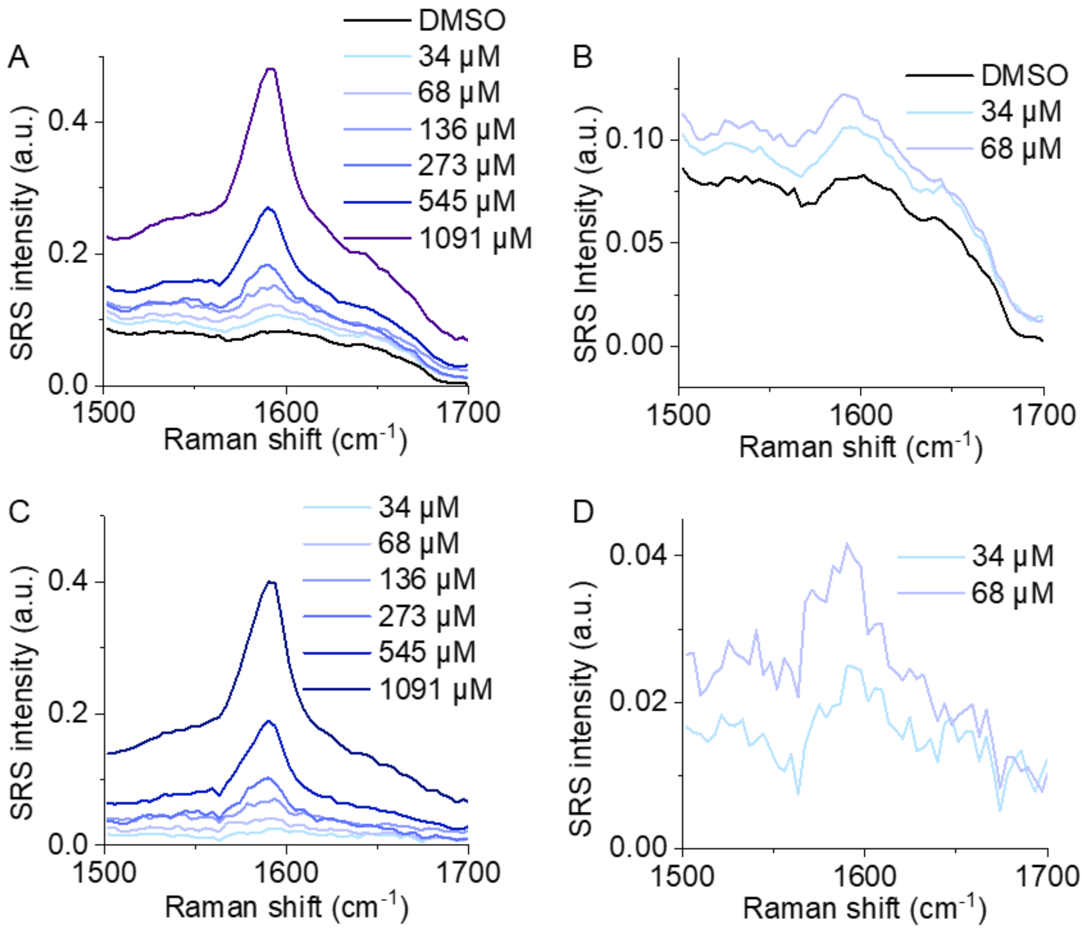

Figure S3. VP-SRS spectra of retinol at different concentrations in DMSO. A) VP-SRS spectral profile of 1091 µM, 545 µM, 273 µM, 136 µM, 68 µM, and 34 µM retinol solution diluted in DMSO. B) Zoom-in VP-SRS spectral profile of 68 µM, 34 µM retinol solution, and pure DMSO solution in (A). C) VP-SRS spectral profile of the raw data in (A) subtracted by DMSO spectral profile. D) Zoom-in VP-SRS spectral profile of 68 µM, 34 µM retinol solution in (C).

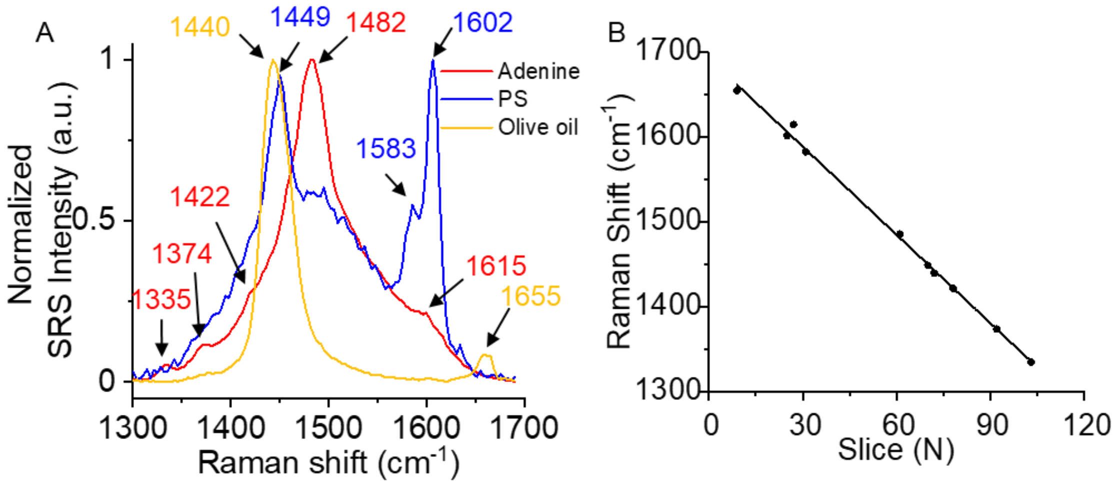

Figure S4. The bandwidth of VP-SRS covers over 300 cm^{-1} in the fingerprint region. A) VP-SRS spectra of adenine, PS beads and olive oil. B) Raman shift is calibrated from moving slice due to the linear relationship between them.

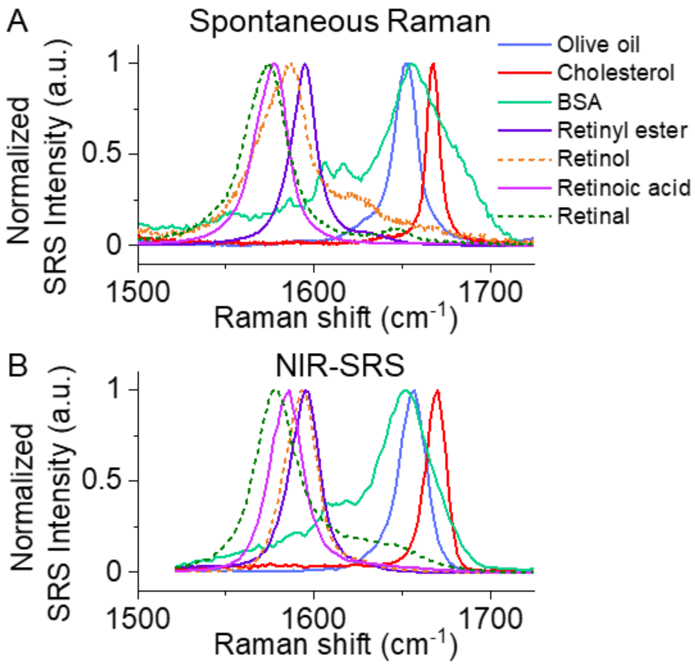

Figure S5. A) Spontaneous Raman and B) NIR-SRS spectra of olive oil, cholesterol, BSA, retinyl ester solution, retinol solution, retinoic acid solution, and retinal solution.

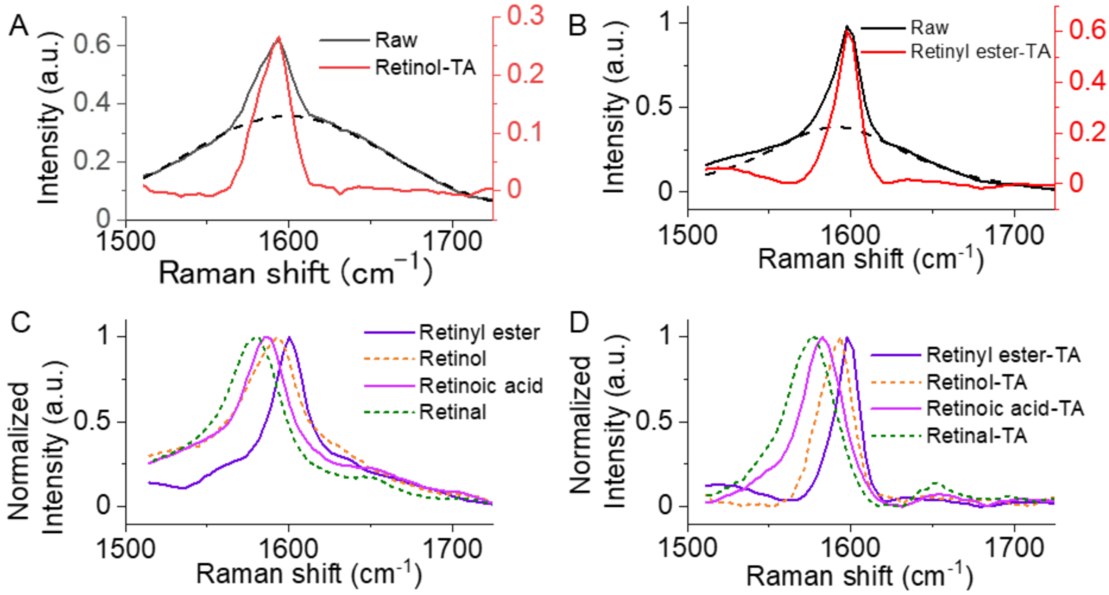

Figure S6. TA-subtracted spectra of retinoids recorded under 522.5-nm Stokes and 484-nm pump. Spectral focusing is at ~1600 cm-1. (A,B) Original spectra (black solid) and fitted TA background (black dash) of (A) retinol and (B) retinyl ester. The TA-subtracted retinoid spectra (red solid) show much narrower FWHM compared with the raw data. (C) Raw and (D) TA-subtracted spectra of retinyl ester (blue solid), retinol (orange dash), retinoic acid (purple solid) and retinal (green dash).

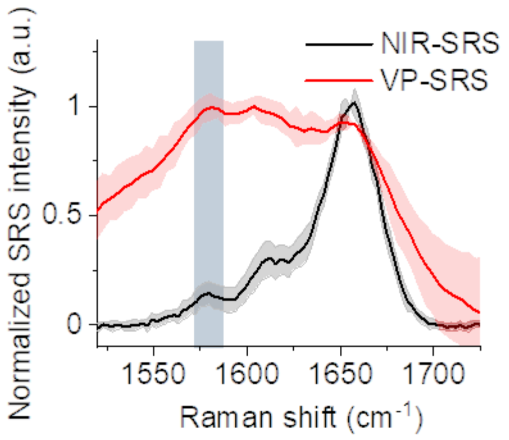

Figure S7. SRS spectra of embryonic neurons detected by NIR-SRS and VP-SRS microscope.

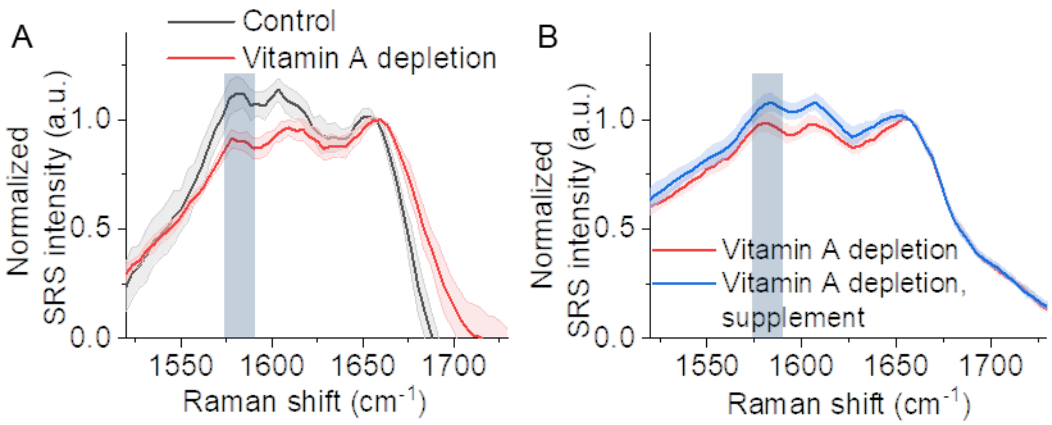

Figure S8. VP-SRS spectra from neuron cells under control, retinol depletion, and rescue.

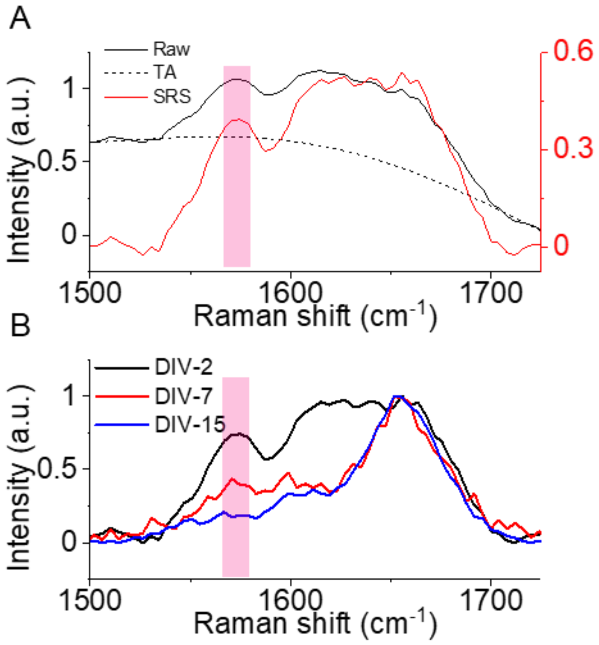

Figure S9. TA-subtracted spectra of retinoids in embryonic neurons recorded under 522.5-nm Stokes and 484-nm pump. Spectral focusing is at ~1600 cm^{-1}. (A) The raw spectrum of a DIV-2 embryonic neuron (black solid), TA background fitting (black dash). The red solid curve shows the SRS spectrum of the DIV-2 embryonic neuron with TA subtracted. (B) SRS spectrum with TA subtracted in DIV-2 (black solid), DIV-7 (red solid) and DIV-15 (blue solid).

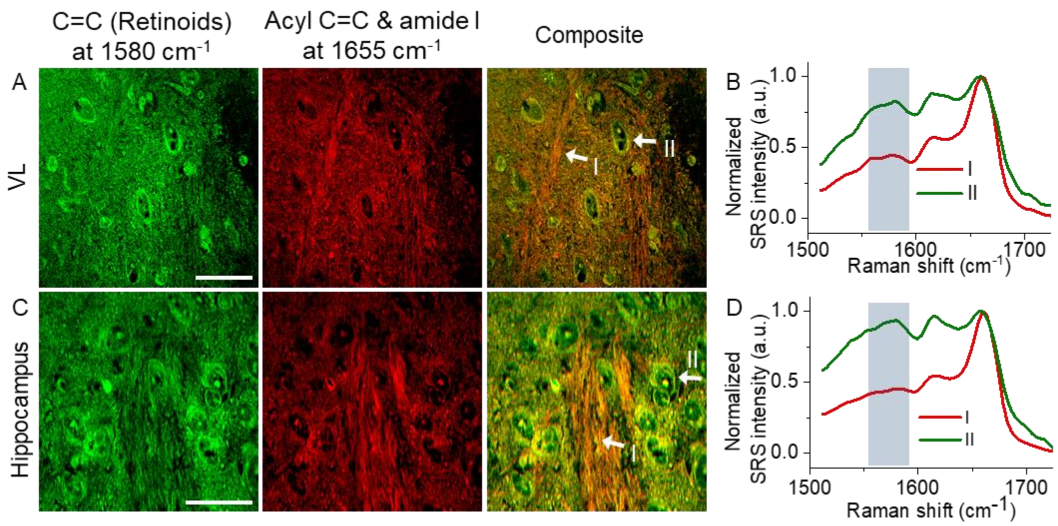

Figure S10. VP-SRS reveals the retinoid distribution in mouse brain. A,C) SRS imaging at 1580 cm^{-1} (left), at 1655 cm^{-1} (middle) and composite (right), respectively, of (A) the ventral lateral nucleus (VL) and (C) hippocampus. (B) and (D) are the corresponding hyperspectra of region I and II indicated in the composite images of (A) and (C), respectively. Scale bar: 20 μm

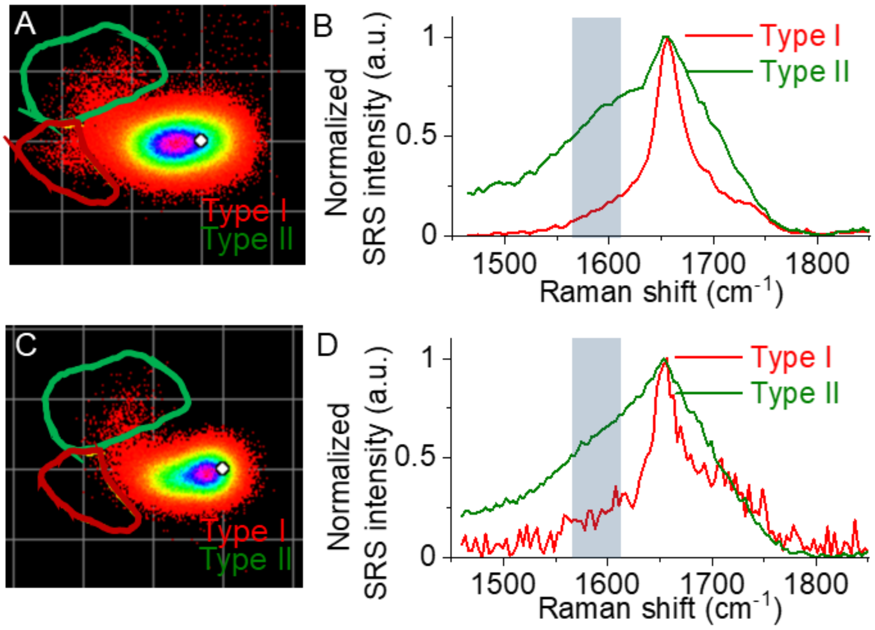

Figure S11. Phasor analysis criteria to differentiate type I and type II LDs in pancreatic cancer cells. A) Phasor plot of the VP-SRS imaging of MIA PaCa-2 cells from Figure 5C. B) Averaged SRS spectrum of type I (red line) and type II (green line) selected by a red-circled region, and by a green-circled region, respectively, in the phasor plot of (A). C) Phasor plot of the VP-SRS imaging of G3K cells from Figure 5C. D) Averaged SRS spectrum of type I (red line) and type II (green line) selected by a red-circled region, and by a green-circled region, respectively, in the phasor plot of (C).

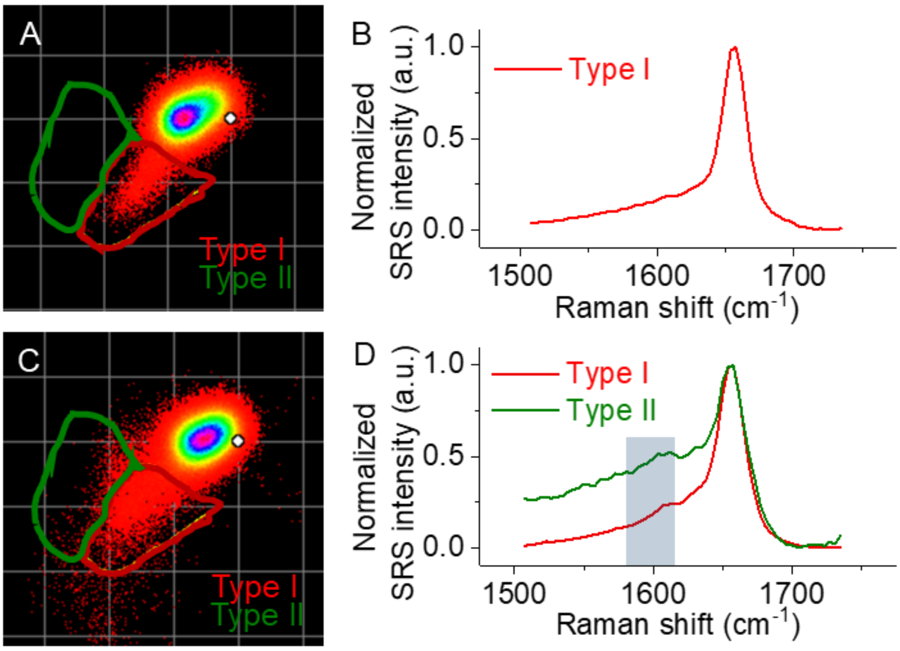

Figure S12. Phasor analysis criteria to differentiate type I and type II LDs in ovarian cancer cells. A) Phasor plot of the VP-SRS imaging of OVCAR cells from Figiure 5E. B) Averaged SRS spectra of type I (red line) LDs selected by a red-circled region in the phasor plot of (A). There is no signal in the green-circled region in the phasor plot of (A). C) Phasor plot of the VP-SRS imaging of OVCAR-cisR cells from Figure 5E. D) Averaged SRS spectra of type I (red line) and type II (green line) LDs selected by a red-circled region, and by a green-circled region, respectively, in the phasor plot of (C).

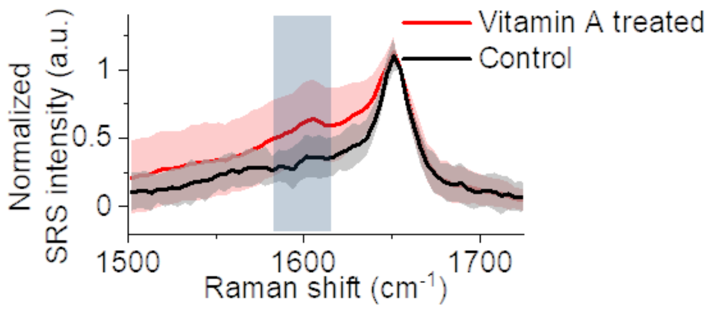

Figure S13. VP-SRS spectra of G3K under control (black solid line) and retinol supplement (red solid line).

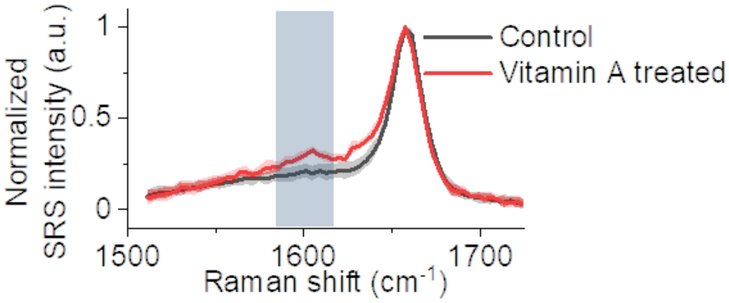

Figure S14. VP-SRS spectra of MIA PaCa-2 under control (black solid line) and retinol supplement (red solid line).

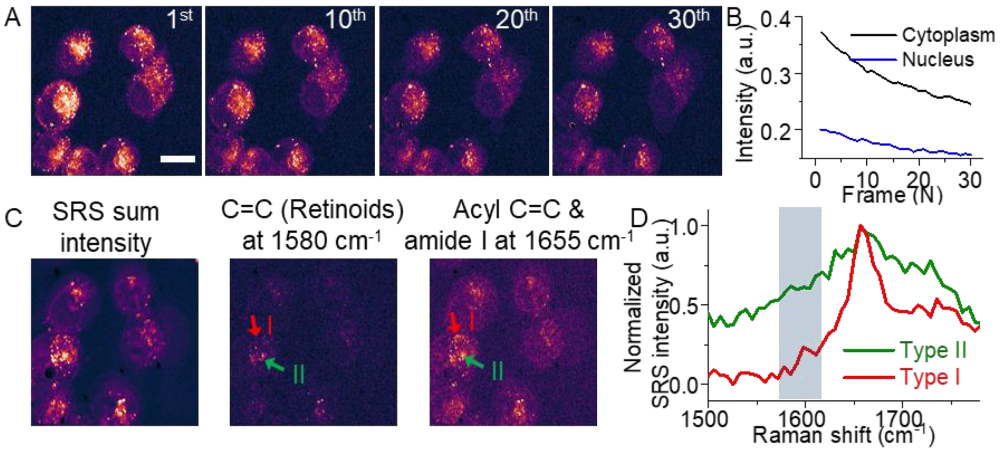

Figure S15. Scanning 30 frames on cells before SRS imaging acquisition was used to get rid of non-SRS signal. A) Bleaching images of G3K pancreatic cancer cells by VP-SRS microscope. VP-SRS microscope induces bleaching of cells especially in cytoplasm when the power of pump and stokes is 30 mW and 40 mW, respectively. Pixel dwell time is 10 µs. B) Bleaching signal in cytoplasm and nucleus were both recorded at different timelapses. C) After 30 frames scanning, SRS imaging of the same G3K cells was acquired successfully, (left) by summing up the SRS signal in the fingerprint region, (middle) at 1655 cm^{-1} corresponding to the mixture of acyl C=C bond and amide I band, (right) at 1580 cm^{-1} corresponding to C=C bond of retinoids. D) SRS spectrum of type I and type II LDs indicated by the white arrows in (C) normalized by signal of the mixture of acyl C=C bond and amide I band at 1655 cm^{-1}. Scale bar: 10 µm.

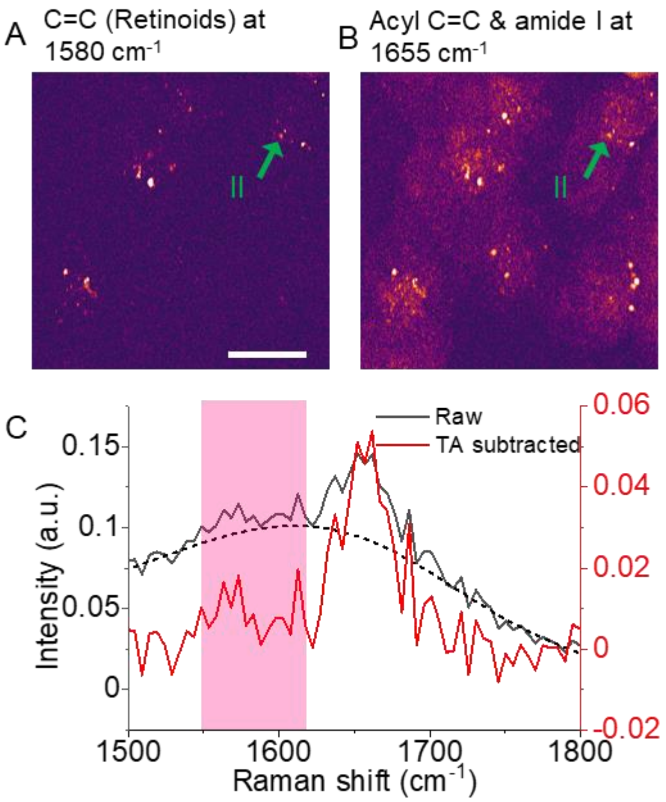

Figure S16. SRS imaging of MIA PaCa-2 cells, (A) at ~1580 cm^{-1} corresponding to C=C bond of retinoids, (B) at 1655 cm^{-1} corresponding to acyl C=C bond and amide I band. (C) The raw spectrum of a type-II LD in a MIA PaCa-2 cell pointed by green arrow in A and B (black solid) and TA background (black dash). The red solid curve shows the SRS spectrum with TA subtracted. Spectral focusing is at ~1570 cm^{-1}. Scale bar: 15 μm.

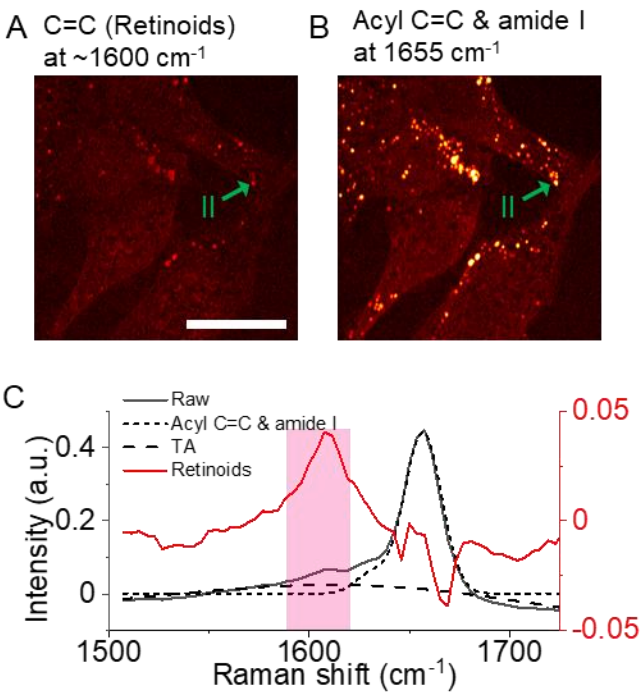

Figure S17. SRS imaging of OVCAR-cisR cells, (A) at ~1600 cm^{-1} corresponding to C=C bond of retinoids, (B) at 1655 cm^{-1} corresponding to acyl C=C bond and amide I band. (C) The raw spectrum of a type-II LD in an OVCAR-cisR cell pointed by green arrow in A and B (black solid), TA background (black dash) and SRS signal contributed by acyl C=C & amide I band at 1655 cm^{-1}(short black dash). The red solid curve shows the retinoid SRS spectrum with TA and 1655 cm^{-1} peak subtracted. Spectral focusing is at ~1600 cm^{-1}. Scale bar: 15 μm.

Part 2: SNR enhancement

We assume that after the objective, the power focused on the sample is the same both under NIR-SRS and VP-SRS.

For VP-SRS, Pump and Stoke wavelength are 482 nm and 522.5 nm, respectively. For NIR-SRS, Pump and Stoke wavelength are 897 nm and 1045 nm, respectively.

SRL can be written as

$$\Delta I_P \propto -N \times \sigma_{Raman} \times I_P \times I_S$$

where ΔI_P denotes the relative loss in the pump laser, N is the number of vibrational bonds in the imaging volume, σ_{Raman} is the Raman scattering cross-section, and I_p, I_S are the laser intensities of the pump and Stokes beam.

Therefore, signal enhancement of VP-SRS compared to NIR-SRS can be written as

$$\frac{\Delta I_{P-VP}}{\Delta I_{P-NIR}} = \frac{N_{VP}}{N_{NIR}} \times \frac{\sigma_{Raman-VP}}{\sigma_{Raman-NIR}} \times \frac{I_{P-VP}}{I_{P-NIR}} \times \frac{I_{S-VP}}{I_{S-NIR}}.$$

1. For N which is the bonds in the focused volumn,

$$\frac{N_{VP}}{N_{NIR}} = \frac{{diameter_{VP}}^3}{{diameter_{NIR}}^3} = \frac{482^3}{897^3} = 0.155.$$

2. For σ_{Raman}, which can express as

$$\sigma_{Raman} = K \times \omega_{pump} \times \omega_{Stokes}^3 \times \left[\frac{\omega_{pump}^2 + \omega_e^2}{(\omega_e^2 - \omega_{pump}^2)^2}\right]^2.$$

Therefore,

$$\frac{\sigma_{Raman-VP}}{\sigma_{Raman-NIR}} = \frac{\omega_{\text{p-VP}} \times \omega_{S-VP}^3 \times \left[\frac{\omega_{\text{p-VP}}^2 + \omega_{\text{e}}^2}{(\omega_{\text{e}}^2 - \omega_{\text{p-VP}}^2)^2}\right]^2}{\omega_{\text{p-NIR}} \times \omega_{\text{S-NIR}}^3 \times \left[\frac{\omega_{\text{p-NIR}}^2 + \omega_{\text{e}}^2}{(\omega_{\text{e}}^2 - \omega_{\text{p-NIR}}^2)^2}\right]^2}$$

$$= \frac{\frac{1}{482} \times \frac{1}{522.5}^3 \times \left[\frac{\frac{1}{482}^2 + \frac{1}{325}^2}{(\frac{1}{325}^2 - \frac{1}{482}^2)^2}\right]^2}{\frac{1}{897} \times \frac{1}{1045}^3 \times \left[\frac{\frac{1}{897}^2 + \frac{1}{325}^2}{(\frac{1}{325}^2 - \frac{1}{897}^2)^2}\right]^2} = 158$$

where, $\omega_e = 325\, nm$ is the center wavelength of retinol absorption (Figure S2, Supporting Information).

3. For I_P and I_s,

$$\frac{I_{P-VP}}{I_{P-NIR}} = \frac{\frac{P}{diameter_{VP}{}^2}}{\frac{P}{diameter_{NIR}{}^2}} = \frac{897^2}{482^2} = 3.46$$

$$\frac{I_{S-VP}}{I_{S-NIR}} = \frac{1045^2}{522.5^2} = 4$$

Therefore,

$$\frac{\Delta I_{P-VP}}{\Delta I_{P-NIR}} = \frac{N_{VP}}{N_{NIR}} \times \frac{\sigma_{Raman-VP}}{\sigma_{Raman-NIR}} \times \frac{I_{P-VP}}{I_{P-NIR}} \times \frac{I_{S-VP}}{I_{S-NIR}} = 0.155 \times 158 \times 3.46 \times 4 = 339.$$

Then for the noise where shot noise dominates and is proportional the photon count, which induces the uncertainty of photocurrent i_{shot}.

The shot noise ratio of VP to NIR is

$$\frac{i_{shot-VP}}{i_{shot-NIR}} = \sqrt{\frac{N_{VP}}{N_{NIR}}} = \sqrt{\frac{482}{897}} = 0.733.$$

In conclusion, SNR should be theoretically enhanced by $\frac{\frac{\Delta I_{P-VP}}{\Delta I_{P-NIR}}}{\frac{i_{shot-VP}}{i_{shot-NIR}}} = \frac{339}{0.733} = 462$ times.
